# Diabetic β-Cells Can Achieve Self-Protection against Oxidative Stress through an Adaptive Up-Regulation of Their Antioxidant Defenses

**DOI:** 10.1371/journal.pone.0006500

**Published:** 2009-08-05

**Authors:** Grégory Lacraz, Florence Figeac, Jamileh Movassat, Nadim Kassis, Josiane Coulaud, Anne Galinier, Corinne Leloup, Danielle Bailbé, Françoise Homo-Delarche, Bernard Portha

**Affiliations:** 1 Equipe B2PE (Biologie et Pathologie du Pancréas Endocrine), Unité BFA (Biologie Fonctionnelle et Adaptative), Université Paris-Diderot et CNRS EAC7059, Paris, France; 2 Equipe HERGE, Unité BFA (Biologie Fonctionnelle et Adaptative), Université Paris-Diderot et CNRS EAC7059, Paris, France; 3 UMR 5241, CNRS and Université P. Sabatier, CHU Rangueil, Toulouse, France; University of Bremen, Germany

## Abstract

**Background:**

Oxidative stress (OS), through excessive and/or chronic reactive oxygen species (ROS), is a mediator of diabetes-related damages in various tissues including pancreatic β-cells. Here, we have evaluated islet OS status and β-cell response to ROS using the GK/Par rat as a model of type 2 diabetes.

**Methodology/Principal Findings:**

Localization of OS markers was performed on whole pancreases. Using islets isolated from 7-day-old or 2.5-month-old male GK/Par and Wistar control rats, 1) gene expression was analyzed by qRT-PCR; 2) insulin secretion rate was measured; 3) ROS accumulation and mitochondrial polarization were assessed by fluorescence methods; 4) antioxidant contents were quantified by HPLC. After diabetes onset, OS markers targeted mostly peri-islet vascular and inflammatory areas, and not islet cells. GK/Par islets revealed in fact protected against OS, because they maintained basal ROS accumulation similar or even lower than Wistar islets. Remarkably, GK/Par insulin secretion also exhibited strong resistance to the toxic effect of exogenous H_2_O_2_ or endogenous ROS exposure. Such adaptation was associated to both high glutathione content and overexpression (mRNA and/or protein levels) of a large set of genes encoding antioxidant proteins as well as UCP2. Finally, we showed that such a phenotype was not innate but spontaneously acquired after diabetes onset, as the result of an adaptive response to the diabetic environment.

**Conclusions:**

The GK/Par model illustrates the effectiveness of adaptive response to OS by β-cells to achieve self-tolerance. It remains to be determined to what extend such islet antioxidant defenses upregulation might contribute to GK/Par β-cell secretory dysfunction.

## Introduction

Oxidative stress (OS) is a well-established mediator of hyperglycemic damage to a wide range of tissues, i.e., neurons, retinal cells and vascular endothelium [1]. Because of their high aerobic glucose metabolism rates, pancreatic β-cells have so far been considered victims of this hyperglycemia-induced reactive oxygen species (ROS) scenario [2], even more than other cell types, as they were reported to express low levels of some classical ROS-scavenging enzyme systems [3], [4].

In human type 2 diabetes, the concept that β-cell dysfunction reflects OS is supported by the following findings: nitrotyrosine and 8-hydroxy-2′-deoxyguanosine (8-OHdG) concentrations are significantly higher in diabetic than control islets and associated with impaired glucose-stimulated insulin secretion (GSIS) [5]; OS-related DNA damage is enhanced [6] and NADPH-oxidase is overexpressed [7] in diabetic pancreases; *in vitro* 24 h exposure of diabetic islets to the antioxidant glutathione decreases the nitrotyrosine concentration [5], suggesting that lowering islet OS might be a potential therapeutic approach to type 2 diabetes.

However, the notion that OS is pro-diabetic is not so straightforward, as evidence from transgene-expressing nonobese diabetic mice studies suggests that antioxidant enzymes e.g., metallothionein and catalase, accelerate spontaneous diabetes [8], highlighting the beneficial role of ROS. Moreover, growing evidence indicates that ROS are involved in signaling normal β-cell–glucose responsiveness [9]–[11]. Hence, to what extent the β-cell is able to react to enhanced and chronic ROS as encountered during type 2 diabetes, remains unknown.

The Goto–Kakizaki/Paris line (GK/Par) of the spontaneous rat type 2 diabetes model is characterized by hyperglycemia and defective GSIS [12]. Pertinently, OS markers such as 8-OHdG and 4-hydroxy-2-nonenal (HNE) were reported high in islets from GK rats (Japanese colony) [13]. Moreover, we previously reported upregulation of pro- and anti-oxidant genes (thioredoxin-interacting protein-1 and glutathione peroxidase-1, respectively), concomitantly with inflammation in diabetic GK/Par islets [14].

This study was designed to evaluate tissue localization of OS markers and the extent to which OS contributes to β-cell injury in GK/Par rats, by exploring the functional susceptibility of diabetic β-cells (insulin secretion) to calibrated ROS exposure, intracellular mechanisms of their adaptation to OS, and time-course of this OS adaptation (taking advantage of GK/Par rat normoglycemia until weaning).

## Results

### OS markers in diabetic GK/Par rats

**Figure 1:**
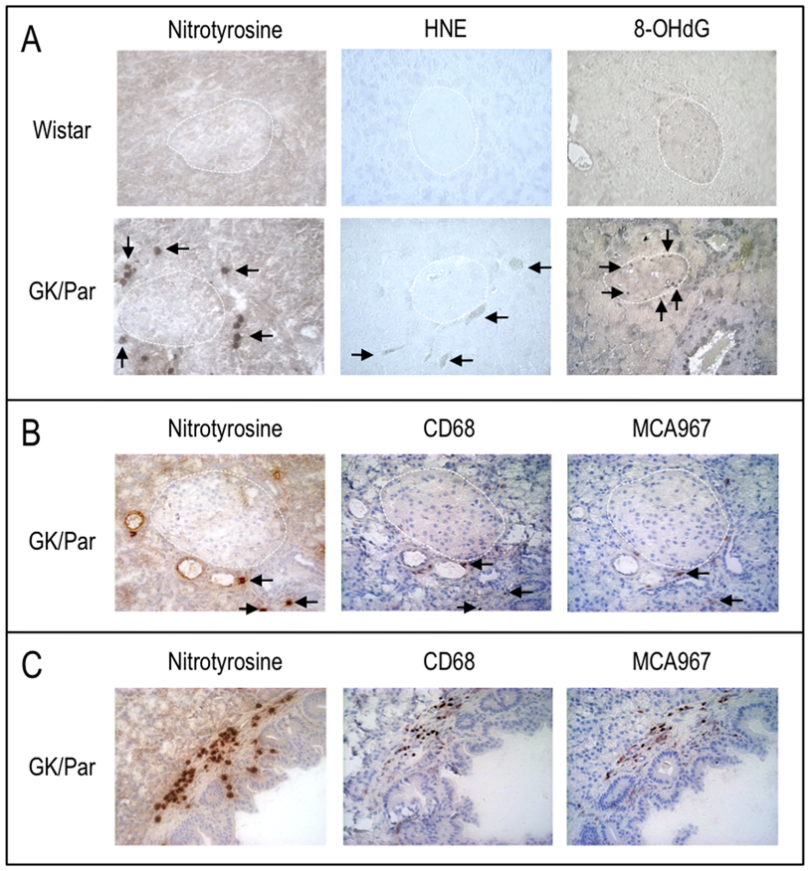
In 2.5-month-old diabetic GK/Par pancreases, nitrotyrosine, 4-hydroxy-2-nonenal (HNE)-modified proteins and 8-hydroxy-2′-deoxyguanosine (8-OHdG) accumulate in the peri-islet vascular and inflammatory compartments. Immunolabelling of nitrotyrosine, HNE-adducts or 8-OHdG (arrows) in pancreatic tissues of GK/Par and Wistar rats (*A*). An islet is encircled in each quadrant. Nitrotyrosine-positive (arrows) material was closely associated with macrophages (CD68) and granulocytes (MCA967) at the islet periphery (*B*), and in the vicinity of pancreatic ducts (*C*). Original magnification×250.

Immunohistochemistry on diabetic GK/Par pancreases (Fig. 1) showed, unlike Wistar, the presence of nitrotyrosine and HNE labelling, which identify ROS and lipid peroxidation, respectively. Marker-positive cells were predominantly localized at the GK/Par islet periphery or along ducts and accompanied inflammatory infiltrates. Such markers were absent in seven-day-old (D7) GK/Par and D7 Wistar pancreases (data not shown).

The glutathione redox state was 14.2% lower (*p*<0.001) in GK/Par red blood cells (RBC) than Wistar (Table 1), with comparable glutathione in its reduced form (Eq GSH) content. In D7 GK/Par RBC, Eq GSH content was half that of D7 Wistar (*p*<0.001), for comparable glutathione redox state. Plasma α-tocopherol was higher in D7 and adult GK/Par than age-matched Wistar rats, indicating that the OS in GK/Par does not result from α-tocopherol deficiency. Altogether, these data showed that signs of overall OS in GK/Par rat developed after diabetes onset.

**Table 1:** Biological characteristics of 7-day-old or 2.5-month-old male Wistar and GK/Par rats.

	7-day-old		2.5-month-old	
	Wistar	GK/Par	Wistar	GK/Par
Body weight (g)	16.0±0.3	9.4±0.2*	384.2±9.7	277.2±8.4*
Plasma glucose (mmol/l)	7.2±0.1	7.0±0.2	5.5±0.1	8.4±0.3*
Plasma insulin (pmol/l)	440±60	160±10*	840±40	1000±80
Plasma α-tocopherol (µmol/l)	25.2±0.9	30.7±1.3*	13.5±0.6	22.0±1.1*
RBC glutathione redox state	92.2±0.6	91.0±0.5	93.5±1.0	80.1±3.1*
RBC Eq GSH content (mmol/l)	71.1±8.2	37.9±3.2*	3.9±0.3	3.8±0.3

Glucose, insulin and α-tocopherol were determined in plasma. Glutathione redox state (% of reduced glutathione (GSH)) and GSH (Eq GSH) content were determined in red blood cells (RBC). Data are means±SEM. Wistar, *n* = 9–13; GK/Par, *n* = 7–9. *p<0.05 *vs.* age-matched Wistar group.

### Resistance of diabetic GK/Par islets to the deleterious ROS effect on insulin secretion

**Figure 2:**
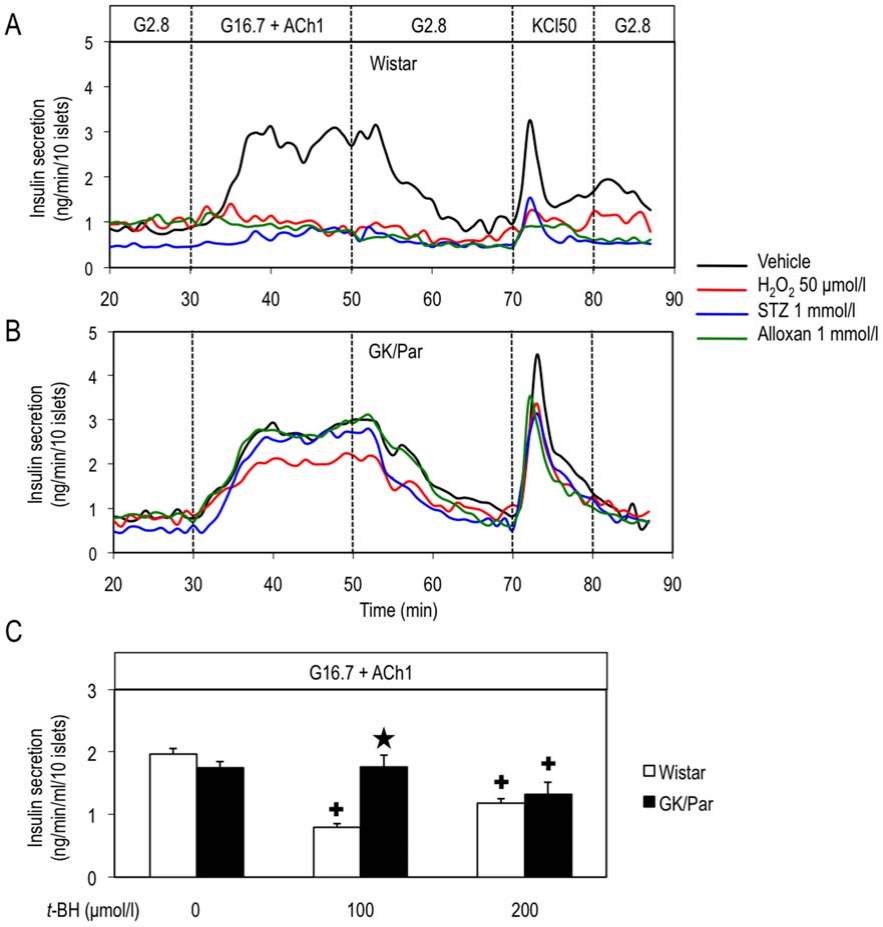
Diabetic GK/Par β-cells are resistant to oxidative stress *in vitro*. Wistar or GK/Par islets were perifused with medium containing (in mmol/l) 2.8 glucose (G2.8), 16.7 glucose+1 acetylcholine (G16.7+ACh1), G2.8, or 50 KCl (KCl50). Vehicle or oxidative agent (H_2_O_2_ 50 µmol/l, or alloxan 1 mmol/l, or streptozotocin (STZ) 1 mmol/l) was added as indicated (*A,B*). ΔIns values (insulin secretion AUC) derived from *panels* (*A*) and (*B*) indicated that GK/Par insulin secretion at G16.7 and KCl50 was strongly resistant to the toxic effects of all oxidative agents used, unlike Wistar. In another set of experiments insulin secretion at G16.7+ACh1 was measured (static incubation, 30 min) in the presence of the GSH-oxidizing agent *tert*-butylhydroperoxide (*t*-BH) at different concentration as indicated (*C*). Data are the means±SEM of 3–6 experiments in each group. *p<0.05 *vs.* age-matched Wistar group; ^+^ *p*<0.05 *vs.* vehicle in the same group.

Acute effects of different ROS-generating agents on GSIS and KCl-stimulated insulin secretion were first evaluated *in vitro*. Because GSIS by GK/Par islets had to be sufficiently high to appreciate a negative ROS impact, we used acetylcholine (ACh) to restore their GSIS [15]. H_2_O_2_ (50 µmol/l), streptozotocin (STZ, 1 mmol/l) or alloxan (1 mmol/l) strongly blunted GSIS by Wistar islets, as assessed by insulin secretion AUC (ΔIns_30–50 min_: −54%, −76%, or −58%, respectively, *p*<0.0001), but none affected GK/Par GSIS (Fig. 2, *panels A and B*). Moreover, the potent oxidant *tert*-butylhydroperoxide (*t*-BH) suppressed GSIS by Wistar islets in a dose-dependent manner, but not in GK/Par islets except at the highest (200 µmol/l) only (Fig. 2C). Therefore, the GK/Par GSIS can be overcome in the presence of elevated ROS concentration.

**Figure 3:**
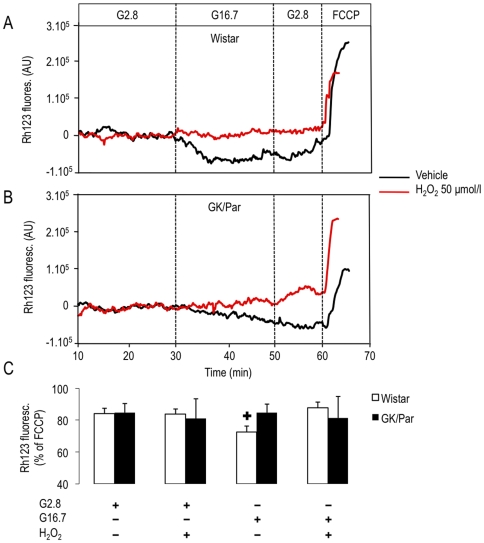
Alteration in mitochondrial membrane potential (ΔΨ_m_) induced by glucose in GK/Par islets. Islets were loaded with rhodamine 123 (Rh123) in KRB–BSA containing G5.5 for 30 min, and the ﬂuorescence intensity was monitored. Representative records of ΔΨ_m_ responses by islets from Wistar (*A*) or GK/Par (*B*) to glucose from 2.8 (G2.8) to 16.7 mmol/l (G16.7), and to carbonyl cyanide *p*-trifluoromethoxyphenylhydrazone (FCCP, 4 µmol/l) that uncouples mitochondria, are shown. Vehicle or oxidative agent (H_2_O_2_, 50 µmol/l) was added as indicated. For each experiment, the Rh123 fluorescence intensity obtained at G2.8 or G16.7 was normalized to the ﬂuorescent intensity obtained after addition of carbonyl cyanide *p*-trifluoromethoxyphenylhydrazone (FCCP, 4 µmol/l) that uncouples mitochondria (*C*). Data are the means±SEM of 3–10 experiments in each group. ^+^ *p*<0.05 *vs.* G2.8.

Given that mitochondria are key players in GSIS [16], changes of mitochondrial hyperpolarization (ΔΨ_m_) in response to high glucose were evaluated. The stimulation of Wistar islets by a glucose change from 2.8 (G2.8) to 16.7 mmol/l (G16.7) induced a 15% decrease in rhodamine 123 (Rh123) ﬂuorescence, demonstrating mitochondrial membrane hyperpolarization (ΔΨ_m_) (Fig. 3A). As expected, addition of carbonyl cyanide *p*-trifluoromethoxyphenylhydrazone (FCCP) rapidly depolarized the mitochondrial membrane to a level that was used as 100% reference for data normalization within each group. In the presence of 50 µmol/l H_2_O_2_, the ΔΨ_m_ was abrogated in Wistar islets (Fig. 3, *panels A and C*). In GK/Par islets, the decrease in Rh123 ﬂuorescence triggered by glucose was abolished (Fig. 3, *panels B and C*) and not further modified by H_2_O_2._

**Figure 4:**
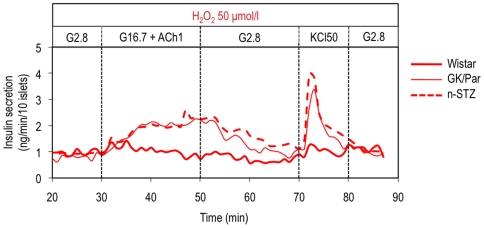
Unlike Wistar β-cells, diabetic neonatally streptozotocin-treated (n-STZ) are resistant to oxidative stress *in vitro*, as GK/Par β-cells. The perifusion experiments are the same that those described in the legend to Fig. 2. Data are the means±SEM of 4–6 experiments in each group.

Finally, to exclude the possibility that our observations were merely an artefact of the GK/Par model, we used the neonatally streptozotocin-treated rat (n-STZ) rat, another type 2 diabetes model [17]. The n-STZ islet secretory responses to ROS were similar to those of GK/Par islets (Fig. 4).

### More AOD in diabetic GK/Par islets

**Figure 5:**
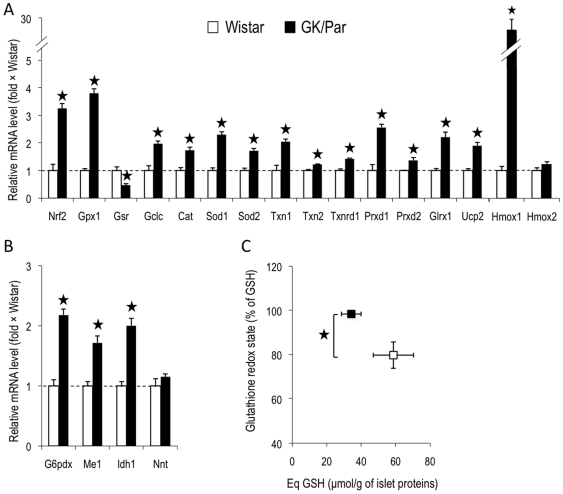
Antioxidant defense status was higher in diabetic GK/Par islets. The mRNA levels for genes encoding antioxidant proteins (*A*). The mRNA levels for genes encoding proteins instrumental in the production of the antioxidant cofactor NADPH (*B*). Intra-insular reduced glutathione GSH (Eq GSH) content was higher in GK/Par than Wistar rats (*C*). Data are the means±SEM of 8–16 experiments in each group. *p<0.05 *vs.* age-matched Wistar group.

The expression of a large set of antioxidant genes involved in ROS detoxification was assessed (Fig. 5, *panels A and B*). The mRNA levels of cytosolic (Sod1) and mitochondrial (Sod2) superoxide dismutases, which represent the first-line defense against superoxide anion generated by the mitochondrial electron transfer chain (ETC), were overexpressed (×2.3, *p*<0.0001; and ×1.7, *p*<0.001, respectively) in GK/Par islets. Similarly, mRNA accumulation was increased for antioxidant enzymes involved in further reduction of superoxide-derived compounds (H_2_O_2_), i.e., catalase (Cat: ×1.7, *p*<0.001), glutathione peroxidase-1 (Gpx1: ×3.8, *p*<0.0001), and enzymes such as thiol/disulfide oxidoreductases, glutaredoxin (Glrx1) (×2.2, *p*<0.001) and thioredoxins (Txn1×2, *p*<0.001; and Txn2×1.2, *p*<0.05). The expression of thioredoxin reductase (Txnrd1), necessary for NADPH-dependent reduction of thioredoxins, was also upregulated (×1.4, *p*<0.01). Peroxiredoxins that catalyze H_2_O_2_ reduction to water using thioredoxins or glutaredoxins as physiological hydrogen donors, were also overexpressed (Prxd1: ×2.6, *p*<0.0001; and Prxd2: ×1.4, *p*<0.05). Finally, the abundance of GSH, the parameter most accurately reflecting the AOD potential, was 19% greater (*p*<0.01) in GK/Par islets (Fig. 5C), and was associated with more γ-glutamylcysteine ligase catalytic subunit (Gclc: ×2, *p*<0.0001) but less glutathione reductase (Gsr: ×0.47, *p*<0.01) mRNAs. Because glutathione reductase regenerates GSH from oxidized glutathione (GSSG), improved redox balance in diabetic GK/Par islets would make this transcript less necessary.

**Figure 6:**
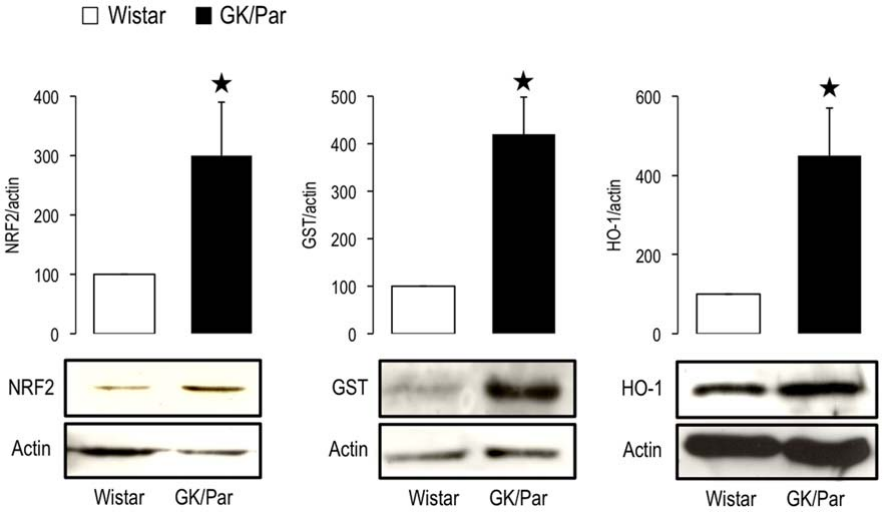
Increased protein expression of some antioxidant defenses in diabetic GK/Par islets. Cell lysates were analyzed by western blot using specific antibody against transcription factor NF-E2–related factor 2 (NRF2), glutathione *S*-transferase (GST), and heme oxygenase-1 (HO-1). Representative blots and proteins quantification are shown. Results are expressed as mean values±SEM for 3 independent experiments with 3–4 animals per experiment. *p<0.05 vs. age-matched Wistar group.

Because NADPH is a major substrate for GSH, thioredoxin and glutaredoxin regeneration, the expression of genes driving NADPH generation were assessed. GK/Par mRNA levels for glucose-6-phosphate dehydrogenase (G6Pdx), malic enzyme-1 (Me1) and isocitrate dehydrogenase-1 (Idh1) were twice Wistar levels (p<0.001), while nicotinamide nucleotide transhydrogenase (Nnt) levels were comparable (Fig. 5B). Thus, NADPH production might be enhanced in GK/Par islets. The gene encoding heme oxygenase-1 (Hmox1), an antioxidant induced by supraphysiological glucose concentrations [18]–[20], was 25-fold overexpressed (*p*<0.0001), with no difference in non-inducible Hmox2 (Fig. 5A). The transcription factor NF-E2–related factor 2 (Nrf2), which drives the expression of many above-mentioned genes [21], [22], was also overexpressed (×3.3, *p*<0.0001) in GK/Par islets. The mRNA levels correlated with a strong increase of the islet NRF2 and HO-1 protein expression as measured by immunoblot (×3 and ×4.5, respectively, *p*<0.05) (Fig. 6). Moreover, the protein level of glutathione *S*-transferase was also higher in these islets (GST: ×4.2, *p*<0.05). GST catalyses the conjugation of GSH to electrophilic centers on a wide variety of substrates and detoxifies endogenous compounds such as peroxidized lipids [23]. Finally the expression of uncoupling protein-2 (Ucp2: ×1.9, *p*<0.0001), which limits superoxide production by dissipating the proton gradient [24], was also increased. All these data strongly suggest that diabetic GK/Par islets are able to protect themselves against ROS toxicity *via* AOD and/or uncoupling, contrarily to naive Wistar islets.

### Less ROS accumulation in diabetic GK/Par islets

**Figure 7:**
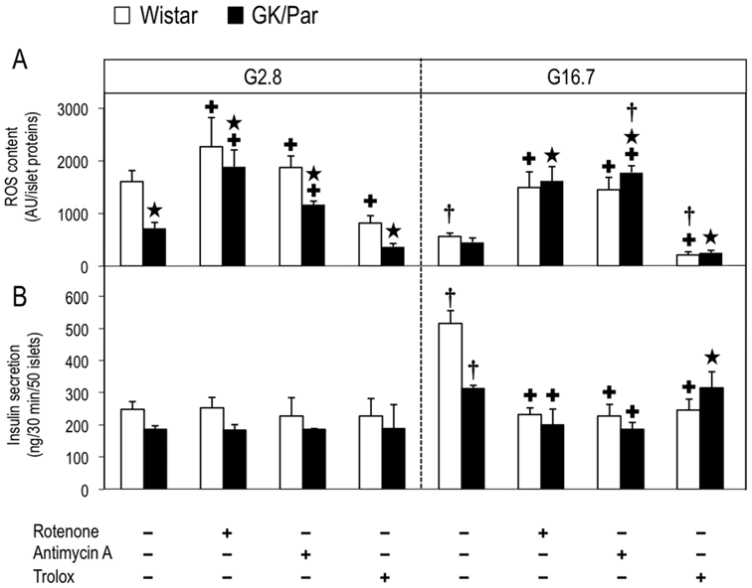
Less reactive oxygen species (ROS) accumulated in diabetic GK/Par than Wistar islets. ROS generation was assessed as CM-H_2_DCFDA fluorescence intensity (arbitrary units (AU)) normalized to total islet proteins in Wistar or GK/Par islets incubated at 2.8 (G2.8) or 16.7 mmol/l glucose (G16.7) (static incubation, 30 min), in the presence or the absence of blockers of the electron transfer chain complexes I (rotenone, 10 µmol/l) or III (antimycin A, 20 µmol/l), or the ROS scavenger (trolox, 1 mmol/l) (*A*). Insulin secretion values derived from experiments (*B*). Data are the means±SEM of 5–23 experiments in each group. *p<0.05 *vs.* age-matched Wistar group; ^+^ *p*<0.05 *vs.* G2.8 or G16.7 for the same group; ^†^ *p*<0.05 *vs.* G16.7 in the same experimental condition.

GK/Par islet AOD upregulation could limit ROS content. Therefore, islet ROS contents, as assessed by 5-(and-6)-chloromethyl-2′,7′-dichlorodihydroﬂuorescein diacetate acetyl ester (CM-H_2_DCFDA), were evaluated over 30 min in response to G2.8 or G16.7. The H_2_O_2_ accumulation by GK/Par islets at G2.8 was half that of Wistar (*p*<0.001) (Fig. 7A). While G16.7 decreased ROS accumulation by 65% (*p*<0.0001) in Wistar (strongest effect from G5.5 to G2.8, data not shown), only a mild decrease was observed in GK/Par islets. At G2.8, rotenone and antimycin A (inhibitors of ETC complexes I and III, respectively) increased ROS contents in Wistar (*p*<0.01) and GK/Par islets (*p*<0.0001) in GK/Par than Wistar islets at both glucose concentrations, suggesting that the low basal ROS content in GK/Par islets did not reflect lower activity of ETC complexes. Concomitantly, these inhibitors blunted GSIS in Wistar (−55%, *p*<0.0001) and GK/Par islets (−48%, *p*<0.05) (Fig. 7B). Trolox, an H_2_O_2-_scavenging water-soluble vitamin E analog, decreased ROS contents in Wistar islets (−57%, *p*<0.05), but had no impact on GK/Par islets, thereby illustrating the inefficacy of antioxidant supplementation in these islets.

### GSH biosynthesis inhibition restores activation of insulin secretion by low H_2_O_2_ level in diabetic GK/Par islets

**Figure 8:**
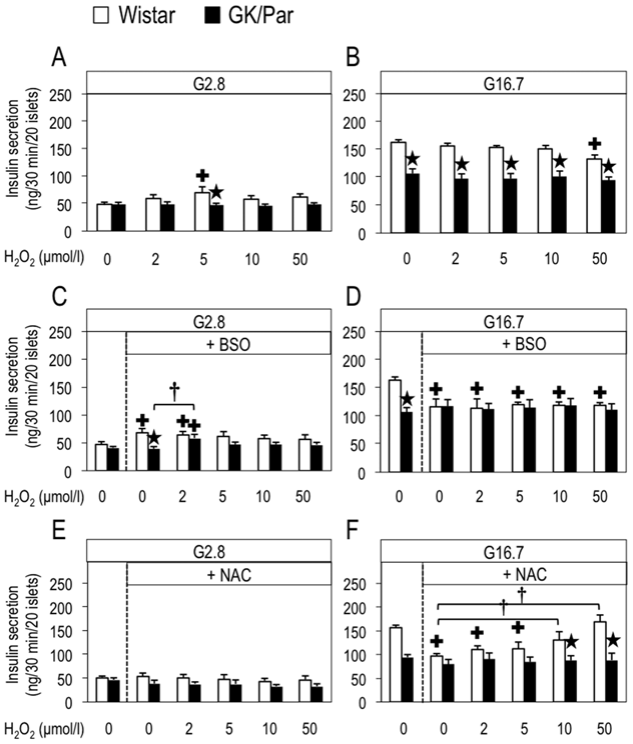
The dual effect (i.e., stimulatory or inhibitory) of exogenous H_2_O_2_ on insulin secretion was abolished in diabetic GK/Par islets. The H_2_O_2_ dose-response effect on insulin secretion at 2.8 (G2.8) or 16.7 mmol/l glucose (G16.7) (static incubation, 30 min) (*A,B*). The same experiments were repeated after pretreatment of islets (1 h) with the reduced glutathione (GSH)-depleting agent buthionine sulfoximine (BSO, 2 mmol/l) (*C,D*) or GSH-inducing agent *N*-acetyl-l-cysteine (NAC, 1 mmol/l) (*E,F*). Data are the means±SEM of 6–23 experiments in each group. *p<0.05 *vs.* age-matched Wistar group;^ +^ *p*<0.05 *vs.* H_2_O_2_ 0 µmol/l in the same group; ^†^ *p*<0.05 *vs.* BSO+H_2_O_2_ 0 µmol/l in the same group. ^†^ *p*<0.05 *vs.* NAC+H_2_O_2_ 0 µmol/l in the same group.

Physiological ROS levels were recently shown to positively signal insulin secretion [9], [11]. Here, we confirmed this mechanism in Wistar islets, as 5 µmol/l H_2_O_2_ significantly increased (50%, *p*<0.05) their insulin secretion at G2.8 (Fig. 8A). By contrast, the ROS-signaling effect was absent in GK/Par islets, but was restored (42%, *p*<0.05) by adding the GSH-biosynthesis inhibitor buthionine sulfoximine (BSO) to 2 µmol/l H_2_O_2_ (Fig. 8C). The ROS-signaling effect in Wistar β-cells was abolished by the GSH-inducer *N*-acetyl-l-cysteine (NAC) (Fig. 8E *vs.* Fig. 8A). In G16.7, NAC decreased GSIS (without H_2_O_2_: −38%, *p*<0.0001) by Wistar but not GK/Par islets (Fig. 8F). The same respective patterns were observed with trolox in Wistar (−53%, *p*<0.001) and GK/Par islets (Fig. 7B). As in the perifusion experiments, GK/Par insulin secretion was resistant to 50 µmol/l H_2_O_2_, unlike Wistar islets (Fig. 8B). Moreover, endogenous ROS levels triggered by GSH depletion either induced by BSO or *t*-BH were less effective in GK/Par when compared to Wistar islets (Fig. 8D and Fig. 2C, respectively). Taken together, these results demonstrated that excess antioxidant (notably GSH) is detrimental for insulin secretion, despite protection against ROS: such a mechanism might operate spontaneously in diabetic GK/Par islets.

### AOD upregulation in GK/Par islets follows diabetes onset

**Figure 9:**
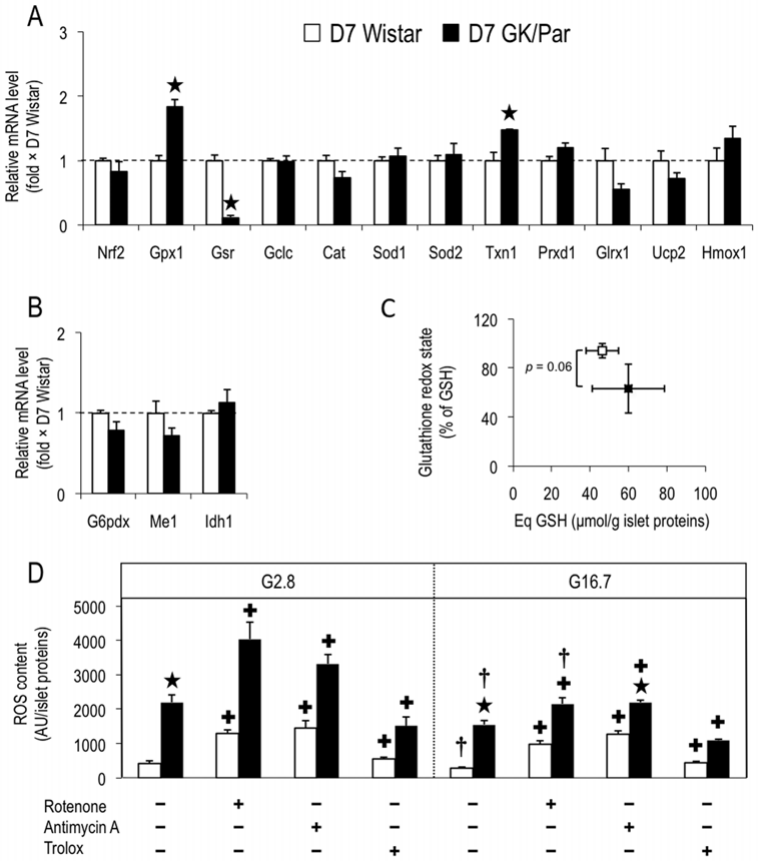
Antioxidant defense status tended to be lower in prediabetic GK/Par rat islets and was associated with enhanced reactive oxygen (ROS) production. Protocols (see the legend to Fig. 5) were repeated with 7-day-old (D7) Wistar or D7 GK/Par rats. The mRNA levels for genes encoding antioxidant proteins (*A*). The mRNA levels for genes encoding proteins instrumental in the production of the antioxidant cofactor NADPH (*B*). Intra-insular reduced glutathione (Eq GSH) content tended to be decreased in D7 GK/Par rats (*C*). ROS generation was assessed as fluorescence intensity (AU) normalized by total islet proteins in D7 Wistar or D7 GK/Par islets incubated for 30 min at 2.8 (G2.8) or 16.7 mmol/l glucose (G16.7), in the presence or the absence of blockers of the electron transfer chain complexes I (rotenone, 10 µmol/l) or III (antimycin A, 20 µmol/l), or the H_2_O_2_ scavenger (trolox, 1 mmol/l) (*D*). Data are the means±SEM of 10–28 experiments (*n* = 15–51 animals) in each group. *p<0.05 *vs.* age-matched Wistar group; ^+^ *p*<0.05 *vs.* G2.8 or G16.7 in the same group; ^†^ *p*<0.05 *vs.* G16.7 in the same experimental condition.

To address the potentially causative role of the diabetic environment in raising AOD, we studied D7 GK/Par islets, because hyperglycemia starts around 1-month of age (weaning) [12]. Since 12 of the 15 selected antioxidant genes were normally expressed in D7 GK/Par islets (Fig. 9, *panels A and B*), we concluded that the enhanced GK/Par AOD was mostly an adaptive response to hyperglycemia-induced OS. However, genes encoding Gsr and Gpx1 were, respectively, underexpressed (×0.12, *p*<0.001) and overexpressed (×1.8, *p*<0.01) in D7 GK/Par islets. This imbalance paralleled a tendency for a lower GK/Par glutathione redox state (−33%, *p* = 0.06), with unchanged Eq GSH content (Fig. 9C), and with a 5-fold increased ROS content, regardless of glucose concentration (*p*<0.0001) (Fig. 9D), compared with age-matched Wistar islets. In Wistar and D7 GK/Par islets, raising glucose from 2.8 to 16.7 mmol/l similarly blunted ROS contents (−33%, *p*<0.05; and −32%, *p*<0.01, respectively) (Fig. 9D). Moreover, at G2.8 or G16.7, ROS contents were increased by rotenone and antimycin A (*p*<0.05), but inhibited by trolox in D7 islets (*p*<0.05). Hence, prediabetic GK/Par islets show elevated ROS levels; AOD upregulation comes later, after diabetes onset.

## Discussion

**Figure 10:**
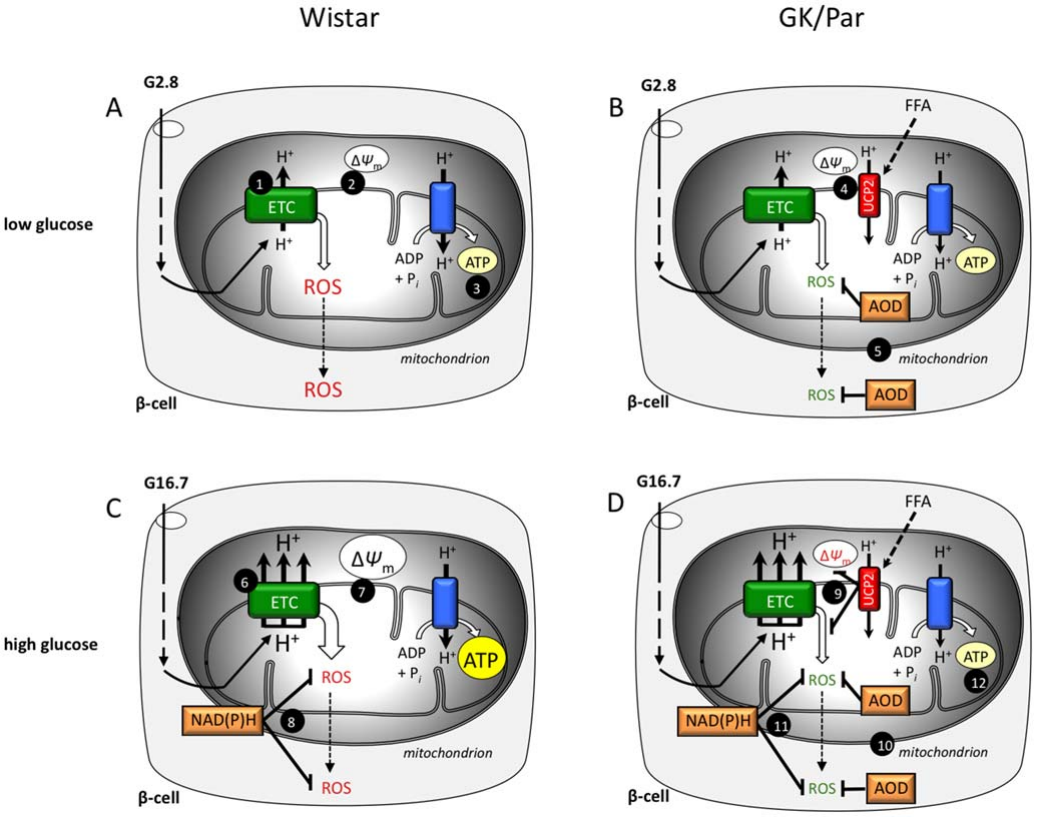
Hypothetical sketch for diabetic GK/Par β-cell adaptive protection against reactive oxygen species (ROS). (*A*) At low glucose (G2.8) in the Wistar β-cell, moderate proton (H^+^) accumulation outside the mitochondrial matrix, which will be passed down the electron transfer chain (ETC) [1], and moderate electrogenic potential (ΔΨ_m_) [2], result from low insulin (ATP) demand [3]. (*B*) In the GK/Par β-cell, ROS accumulation is lower because of: i) uncoupling protein-2 (UCP2), which dissipates ΔΨ_m_ [4] and may be activated by FFA; and ii) antioxidant defense systems (AOD) in mitochondria and cytosol [5], both of which guarantee β-cell self-protection against oxidative damage. (*C*) At high glucose (G16.7) in the Wistar β-cell, glycolytic flux is elevated, resulting in the accumulation of H^+^, and acceleration of electron transport [6]. As a result, the ΔΨ_m_ rises up to a point where its electrogenic potential tends to overcome the driving force of respiration-driven proton pumps (hyperpolarization) [7]: at this stage, ETC slows down, electrons accumulate, and more “random” single electron transfer reactions from ETC components to molecular oxygen enhance ROS generation. However, high glucose metabolism, through elevated NAD(P)H generation [8], improves the ROS-scavenging potential, resulting in low ROS accumulation. (*D*) In the GK/Par β-cell, ROS accumulation remains low due to UCP2 [9], AOD [10] and NAD(P)H [11]. As a consequence however, heightened AOD and/or mitochondrial uncoupling might directly weaken the GK/Par β-cell secretory function when exposed to high glucose because of insufficient ATP generation [12].

The results of this study show that diabetic GK/Par pancreases accumulate markers of OS. This accumulation was most prominent in non-endocrine peri-insular cells near GK/Par islets, and was not observed in endocrine cells within the core of the islets. For the first time, we demonstrated that GK/Par islet cells develop an unexpected adaptive protection to counteract chronic OS, since they were able to maintain basal ROS accumulation lower than in non-diabetic islet cells, due to induction of AOD. It is hypothesized that such islet AOD upregulation might contribute to GK/Par β-cell secretory dysfunction (Fig. 10).

### Enhanced OS within diabetic GK/Par pancreases

As in diabetic patients [2], [6], GK/Par rats exhibited systemic OS, reflected by decreased glutathione redox status in RBC. Moreover, immunohistochemistry revealed that some non-endocrine peri-insular cells within the GK/Par pancreases accumulated oxidative products. While no HNE-adduct was detectable in prediabetic D7 GK/Par pancreases, HNE labelling was located close to vascular areas in diabetic GK/Par pancreases, as previously described in *db/db* mice [25]. The age-related accumulation of 8-OHdG and HNE-modified proteins was reported to correlate to hyperglycemia duration in the GK rat pancreases (Japanese colony) [13]. Macrophages and granulocytes infiltrate around/within GK/Par islets after diabetes onset [14]. Some nitrotyrosine-positive cells were found among inflammatory infiltrates in diabetic GK/Par pancreases, while such cells were undetectable in D7 GK/Par pancreases (data not shown). Interestingly, similar localization within/around islets was also described in NOD mouse [26] and type 2 diabetic humans [27], and it is known that pro-inflammatory cytokine activities, like those of IL-1β and TNF-α, involve ROS generation [28].

### Resistance of diabetic GK/Par islets to ROS-induced suppression of GSIS and enhanced AOD

ecause our data indicated an OS environment surrounding diabetic GK/Par β-cells, the acute effects of several oxidative stressors were tested *in vitro*. None of them had an effect on GK/Par GSIS, unlike Wistar. This remarkable ability to resist against deleterious ROS entails a heightened ability to dissipate free radical-induced stress. The effect of BSO, an inhibitor of GSH biosynthesis, upon GSIS, was also investigated. As expected, BSO, which lowers intra-islet GSH levels and raises endogenous islet peroxide [29], diminished Wistar GSIS, but had no effect in GK/Par, strongly suggesting higher AOD. Because the weak antioxidant status of normal β-cells has been considered a major feature of their poor resistance to oxidative injury [3], [4], stimulating AOD is currently considered the best way to strengthen β-cell resistance to ROS [2], [4], [5], [30]–[33]: this is what we found in GK/Par islets. First, their islet glutathione was entirely in the reduced state, this reflecting a lack of OS in the cellular hydrophilic compartment. Second their high islet GSH level was associated with enhanced Gclc mRNA. This is consistent with the report that Gclc overexpression increased intra-islet GSH and partially prevented the GSIS decrease caused by ROS generated after islets exposure to IL-1β [28]. Once synthesized, GSH serves as a substrate for glutathione peroxidase and GST, which destroy H_2_O_2_, lipid peroxides and peroxinitrite [23]. Accordingly, high Gpx1 mRNA in GK/Par islets might protect islet cells against ROS [29] and limit nitrotyrosine (peroxinitrite-reaction product) formation.

Concomitantly, GK/Par islets strongly express a wide battery of crucial antioxidant genes that could contribute to the very low GSSG level. Indeed, the best protection against ROS appears to require combined overexpressions of genes encoding superoxide-inactivating isoenzymes and H_2_O_2_-inactivating enzymes (Gpx1 and catalase) [30]–[32]. This combination was especially effective in mouse islets because it protected mice against STZ-induced diabetes and limited islet nitrotyrosine accumulation [33], a pattern similar to that observed in GK/Par islets. Genes encoding for thioredoxin metabolism-related proteins and Hmox1, known to be induced in β-cells during OS [18], [19], [22], [35]–[37] and protective under stressful conditions [36], [37], might also be at work in GK/Par islets. Finally, globally enhanced AOD in GK/Par islets might partly reflect the overexpression of Nrf2, a key player in the regulation of genes encoding NADPH-generating enzymes and antioxidant response genes encoding Gclc, Trx, Hmox1 and Gst [21], [22].

### Decreased GSIS and adaptive AOD in diabetic GK/Par islets

It is generally thought that the pathway activating insulin secretion sharply enhances endogenous ROS production, and high glucose stimulation concurs with elevated ROS production in several β-cell/islet models [9], [29], [38]. By contrast, we, like Martens et al. [39], found that increasing glucose to 16.7 mmol/l did not raise ROS contents in Wistar islets, but rather decreased them. The contrast between these observations and data indicating the primary role of ROS generation in chronic hyperglycemia-associated β-cell dysfunction/death is intriguing and remains unexplained. Our data clearly showed that endogenous ROS accumulation in diabetic GK/Par islets was lower than in prediabetic islets and it remained unchanged in response to acute high glucose. Suppression of β-cell mitochondrial ROS formation could potentially be contributory: however, this possibility is unlikely because our data show an increased ROS accumulation in the presence of mitochondrial complex blockers in diabetic GK/Par as well as in normal islets. High rates of glucose metabolism do not increase but prevent ROS accumulation in primary β-cells, an effect that is more pronounced in β-cells with a higher metabolic responsiveness to glucose [39]. Conversely, low glucose concentrations lead to a sustained ROS production in β-cells [40]. Because modestly decreased, normal or even slightly increased glycolytic and mitochondrial glucose oxidation rates have been reported in diabetic GK/Par rats [41]–[44], and as we found normal glucose oxidation rate in GK/Par islets in parallel experiments (Fradet, unpublished data), it seems improbable that decreased ROS accumulation relies on major changes in glucose sensing and metabolism. The most likely mechanism by which ROS production is blunted in diabetic GK/Par islets is *via* their raised AOD (Fig. 10).

The relationship between the ROS-induced increase of β-cell AOD and the impaired insulin secretion is questionable since increased β-cell ROS production could lead to glucose-sensor (glucokinase) inhibition [45] or suppression of glyceraldehyde-3-phosphate dehydrogenase activity [46], with impaired GSIS in both cases. These options do not apply in fact to GK/Par islets, because their glucose-activated glycolytic flux was normal or enhanced [41], [42, Fradet, unpublished data]. A more attractive hypothesis would be that defective GK/Par GSIS results from mitochondrial uncoupling [47]. That concept is supported by our findings of Ucp2 overexpression and lack of ΔΨ_m_ change in response to high glucose, along with the reported lower ATP/ADP ratio in GK/Par islets [48]. This hypothesis is also consistent with the established uncoupling-dependent mechanism of protection against OS as being responsible for diminished ATP production, leading to impaired GSIS [20], [45], [49], [50] (Fig. 10). Finally, our observation that ROS sensing can be obtained when GK/Par islets are challenged, with low H_2_O_2_ concentrations in the presence of BSO (as are the Wistar islets in the absence of BSO), also supports the notion that the enhanced AOD are detrimental for insulin secretion.

### Adaptive increase of AOD and chronic diabetic environment in GK/Par islets

As previously mentioned, after diabetes onset in GK/Par rats, β-cells are exposed *in vivo* to chronic inflammation and therefore to a complex combination of ROS/NO/cytokines [51], [52], first at the islet periphery before progressing into the islets [14]. We hypothesize that type 2 diabetic islet inflammation in GK/Par rats might have originated from endothelial cell (EC) activation [53]. While hyperglycemia itself is well recognized to be deleterious for EC, other factors, such as hyperlipidemia and insulin resistance, which can precede hyperglycemia, might also be able to mediate EC in GK/Par islets [1], [52]. Also nitrotyrosine-/HNE-modified protein accumulation in peri-islet structures, inducible NO synthase overexpression, together with nuclear factor-kappa B subunit p65 transactivation (data not shown) and overexpression of various stress genes (especially Hmox1), were time-correlated with diabetes exposure. Consistent with the GK/Par scenario, exposure of INS-1 cells or mouse islets to HNE markedly increased AOD, including mRNA of Nrf2-targeted genes, and depressed GSIS [9]. Moreover, chronic hyperglycemia is known to be a major cause of islet OS, as assessed by HNE and nitrotyrosine markers in diabetic mice [25], [26]. Furthermore, islet mRNA levels of some stress/AOD genes were enhanced by supraphysiological glucose levels *in vitro* [18] or *in vivo* in pancreatectomized rats, but were reversed after glycemia normalization [19], [20]. The GK/Par islet model fits well with these models of OS adaptation, as it shows upregulation of most stress genes and antioxidant molecule (GSH) after diabetes onset only.

However, the contribution of the prediabetic period is certainly also crucial for adaptation. Indeed, we recently observed, in prediabetic D7 GK/Par sera, increased FFA levels and a high cholesterol/HDL ratio, together with elevated levels of chemokines, which were probably of vascular origin [53]. In addition, FFA-induced ROS production was at play in situations triggering lipid overload in β-cells [54]. Taken together, an inflammatory atherosclerotic-like reaction could explain the high levels of ROS as well as alterations in glutathione and thioredoxin-related gene expression in GK/Par islets at this age. It is possible that this microangiopathy/atherosclerosis islet process might be initiated by a dyslipidemia during the prenatal period because higher circulating cholesterol/HDL ratio is already present in E21.5 GK/Par fetuses and would be instrumental in *in utero* programming of islet endothelial activation/OS in the GK/Par rat [53].

Finally, the low islet functional susceptibility to ROS exposure we found in another type 2 diabetes model, the n-STZ-treated rat, strengthens the possibility that chronic hyperglycemia promotes β-cell self-adaptation to OS. In conclusion, our study shows that the possibility for eukaryotic cells to acquire tolerance to lethal ROS doses by prior exposure to sublethal doses [55], [56], is also operative under pathological conditions such as spontaneous type 2 diabetes.

## Materials and Methods

All animal experiments were conducted on age-matched male GK/Par [12] and non-diabetic Wistar rats from our local colonies in accordance with accepted standards of animal care, established by the French National Center for Scientific Research (CNRS). In some experiments, newborn Wistar received STZ to induce type 2 diabetes [17]. All pharmacological agents, except ^125^I-labeled insulin (DiaSorin) and metaphosphoric acid (VWR Prolabo), were purchased from Sigma-Aldrich.

### Immunohistochemistry

For HNE and 8-OHdG labelling, Wistar and GK/Par rat pancreases were fixed in aqueous Bouin's solution and embedded in paraplast, according to standard procedures. For nitrotyrosine labelling, pancreases were frozen in *n*-hexane on dry ice-chilled alcohol [14]. Immunohistochemistry used: 1) rabbit anti-mouse nitrotyrosine (Upstate) and anti-Ig horseradish peroxidase detection kit (BD Pharmingen) according to the manufacturers' instructions; 2) mouse anti-HNE or 3) mouse anti-8-OHdG (the last two antibodies from Japan Institute for the Control Aging), the secondary antibody (biotin-conjugated rabbit F(ab′)2 anti-mouse IgG, Serotec), and the Vectastin Universal ABC-AP kit (Vector), followed by the Vector Black substrate kit. CD68 and MCA967 labellings were done as described by the manufacturer.

### Islet isolation

D7 or non-fed (post-absorbtive state) 2.5-month-old adult rats were killed by decapitation and pancreatic islets were isolated in collagenase (Sigma) and then handpicked under a stereomicroscope [15].

### Insulin secretion

Freshly isolated islets were perifused as previously described [15] and exposed to G2.8 or G16.7 or KCl 50 mmol/l (KCl50), in the absence or presence of oxidant (50 µmol/l H_2_O_2_, or 1 mmol/l STZ, or 1 mmol/l alloxan) for times indicated. For static incubations, batches of 10, or 20, or 50 freshly isolated islets were preincubated with 1 mmol/l NAC or 2 mmol/l BSO for 1 h at 37°C in 1 ml Krebs–Ringer bicarbonate (KRB)–BSA containing 5.5 mmol/l glucose (G5.5). After washing, pretreated islets were stimulated for 30 min at 37°C in 1 ml KRB–BSA containing G2.8 or G16.7 alone or supplemented with H_2_O_2_ or *t*-BH at different concentrations. A radioimmunoassay determined insulin secretion.

### Mitochondrial membrane potential (ΔΨ_m_)

Rh123 (Invitrogen) fluorescence (excitation: 488 nm; emission: 530 nm) was used as an indicator of ΔΨ_m_ as described [10], [57]. This ﬂuorescent lipophilic cationic dye is known to be specifically partitioned into negatively charged mitochondrial membranes. In cells preloaded with Rh123, when ΔΨ_m_ increases (hyperpolarization) as seen after nutrient stimulation, more Rh123 is concentrated into the mitochondrial membrane, leading to an aggregation of dye molecules and quenching of the ;ﬂuorescence signal. Depolarization of ΔΨ_m_, on the other hand, allows dye to redistribute from mitochondria into the cytosol, resulting in an increase of the Rh123 signal. In the present study, intact islets were loaded with Rh123 (10 µg/ml) for the last 20 min of a 1-h preincubation in KRB–BSA containing G5.5. Then, 10 islets were perifused on the stage of an inverted fluorescent microscope (Nikon Diaphot). After background subtraction, the ﬂuorescence emitted from each islet was normalized to the ﬂuorescence level measured after addition of FCCP (4 µmol/l) that uncouples mitochondria.

### ROS measurements

Islets were loaded with the fluorescent probe CM-H_2_DCFDA (4 µmol/l) (Invitrogen) for 30 min at 37°C [10] and then rapidly frozen and stored at −80°C. Supernatant ROS content (200 µl) was quantified with a reader (Fluostar Galaxy, BMG; excitation: 485 nm; emission: 530 nm).

### mRNA analysis

Total RNA was isolated from islets using the RNeasy mini kit (Qiagen) and its concentration determined by optical density at 260 nm. To remove residual DNA contamination, the RNA samples were treated with RNase-free DNAse (Qiagen) and purified with RNeasy mini-column (Qiagen). Total RNA (4 µg) from each islet sample was reverse transcribed with 40 U of M-MLV Reverse Transcriptase (Invitrogen) using random hexamer primers. The primers used were derived from rat sequences and designed using OLIGO6. Real-time quantitative PCR amplification reactions were carried out in a LightCycler 1.5 detection system (Roche) using the LightCycler FastStart DNA Master plus SYBR Green I kit (Roche). Reverse transcribed RNA (10 ng) was used as the template for each reaction. All reactions were run in duplicate with no template control. The PCR conditions were: 95°C for 10 min, followed by 40 cycles at 95°C for 10 s, 60°C for 10 s and 72°C for 10 s. mRNA transcript levels of 4 housekeeping genes (rpL19, Tbp, cyclophilin a, 18S) were assayed; rpL19 was retained for normalization of other transcripts.

### Western blot analysis

Western blot analysis was performed as previously described [58] to study NRF2, or GST, or HO-1 protein expression. Briefly, 25 µg (for GST and HO-1) or 50 µg (for NRF2) of proteins extracted from GK/Par or Wistar islets were subjected to SDS-PAGE (10% acrylamide gel) and then transferred to a PVDF membrane for 2 h (120 V) using a Bio-Rad Mini Trans Blot electrophoretic transfer unit (Bio-Rad). The membranes were blocked for nonspecific binding with 5% non fat dry milk in Tris-buffered saline (TBS, 20 mM Tris-HCl, 150 mM NaCl, pH 7.4) supplemented with 0.05% Tween 20 (TTBS) and then probed with the specific primary antibodies. After 3 washes with TTBS, membranes were incubated with appropriate horseradish peroxidase-conjugated secondary antibodies. Separated proteins were visualized by an ECL kit (GE Healthcare) and light emission was captured on X-ray film (GE Healthcare). Intensities of the respective bands were examined by densitometric analysis (Scion Image Analyst program).

### Glutathione and α-tocopherol measurements

To measure glutathione, RBC (50 µl) were mixed with 1% EDTA/5% metaphosphoric acid (1∶5 v/v; 450 µl), and 100 or 200 islets were mixed with 5% metaphosphoric acid (300 µl). After centrifugation (3000 *g*, 10 min, 4°C), GSH and GSSG were identified in supernatants by reverse-phase HPLC with electrochemical detection [59]. Results are expressed as mmol/l of RBC or µmol/g of islet proteins. Total glutathione content, referred to as “equivalent GSH” (Eq GSH), is the sum of GSH and doubled GSSG concentrations (2GSH→GSSG). The glutathione redox state is: ([reduced form]/[total forms])×100, with [total forms] = [oxidized form]+[reduced form]. α-Tocopherol was determined, as above, in heparinated plasma (100 µl) extracted with 2-propanol (400 µl) [60]. Results are expressed as µmol of plasma.

### Data presentation and statistical analysis

Variations of ΔΨ_m_ are presented as fluorescence changes over the basal level (G2.8). Integrated elevation above baseline value (ΔIns (AUC for insulin secretion), in ng/10 islets) was used to quantitate insulin response. Data are presented as means±SEM. Statistical analyses used an unpaired Student's *t* test or ANOVA as appropriate. Significance was defined as *p*<0.05.
